# *Lactobacillus crispatus* M247 oral administration: Is it really an effective strategy in the management of papillomavirus-infected women?

**DOI:** 10.1186/s13027-022-00465-9

**Published:** 2022-10-21

**Authors:** Miriam Dellino, Eliano Cascardi, Antonio Simone Laganà, Giovanni Di Vagno, Antonio Malvasi, Rosanna Zaccaro, Katia Maggipinto, Gerardo Cazzato, Salvatore Scacco, Raffaele Tinelli, Alessandro De Luca, Marina Vinciguerra, Vera Loizzi, Antonella Daniele, Ettore Cicinelli, Carmine Carriero, Chiara Antonia Genco, Gennaro Cormio, Vincenzo Pinto

**Affiliations:** 1 Department of Biomedical Sciences and Human Oncology, Policlinic of Bari, University of Bari, Piazza Aldo Moro, 70100 Bari, Italy; 2 Clinic of Obstetrics and Gynecology, “San Paolo” Hospital, ASL Bari, Bari, Italy; 3 Department of Medical Sciences, University of Turin, Turin, Italy; 4 Pathology Unit, FPO-IRCCS Candiolo Cancer Institute, Candiolo, Italy; 5 Unit of Gynecologic Oncology, ARNAS “Civico—Di Cristina—Benfratelli”, Department of Health Promotion, Mother and Child Care, Internal Medicine and Medical Specialties (PROMISE), University of Palermo, 90127 Palermo, Italy; 6 Department of Emergency and Organ Transplantation, Pathology Section, University of Bari “Aldo Moro”, Piazza Giulio Cesare 11, 70124 Bari, Italy; 7 Department of Basic Medical Sciences and Neurosciences, University of Bari “Aldo Moro”, Piazza Giulio Cesare 11, 70124 Bari, Italy; 8 Department of Obstetrics and Gynecology, “Valle d’Itria” Hospital, Martina Franca, Italy; 9 Gynecologic Oncology Unit, IRCCS Istituto Tumori Giovanni Paolo II, Department of Interdisciplinary Medicine (DIM), University of Bari “Aldo Moro”, 70121 Bari, Italy; 10 Institutional BioBank, Experimental Oncology and Biobank Management Unit, IRCCS Istituto Tumori Giovanni Paolo II, Bari, Italy; 11 Departmental of Cervical-Carcinoma Screening, ASL Bari, 70121 Bari, Italy

**Keywords:** HPV infection, *Lactobacillus crispatus* M247, Probiotics, Papillomaviridae, Microbiota, Uterine cervical neoplasms

## Abstract

**Background:**

Recent studies have shown the importance of the microbiota in women's health. Indeed, the persistence of Human Papilloma Virus (HPV)-related lesions in patients with dysbiosis can be the antechamber to cervical cancer. The aim of this study was to evaluate whether long term administration of oral *Lactobacillus crispatus* can restore eubiosis in women with HPV infections and hence achieve viral clearance.

**Methods:**

In total, 160 women affected by HPV infections were enrolled at the Department of Gynecological Obstetrics of “San Paolo” Hospital, Italy between February 2021 and February 2022. The women were randomly assigned to two groups, one in treatment with oral *Lactobacillus crispatus* M247 (group 1, n = 80) versus the control group, that hence only in follow-up (Group 2, n = 80).

**Results:**

After a median follow-up of 12 months (range 10–30 months), the likelihood of resolving HPV-related cytological anomalies was higher in patients in treatment with the long term oral probiotic (group 1) versus the group that perfom only follow-up (group 2) (60.5% vs. 41.3%, *p* = 0.05). Total HPV clearance was shown in 9.3% of patients undergoing only follow-up compared to 15.3% of patients in the group taking long term oral *Lactobacillus crispatus* M247 (*p* = 0.34). However, the percentage of HPV-negative patients, assessed with the HPV-DNA test, documented at the end of the study period was not significantly different from the control group.

**Conclusions:**

Despite the limitations of our analysis, we found a higher percentage of clearance of PAP-smear abnormalities in patients who took long term oral *Lactobacillus crispatus* M247 than in the control group. Larger studies are warranted, but we believe that future research should be aimed in this direction.

*Trial registration* This study is retrospectively registered.

## Introduction

**Fig. 1:**
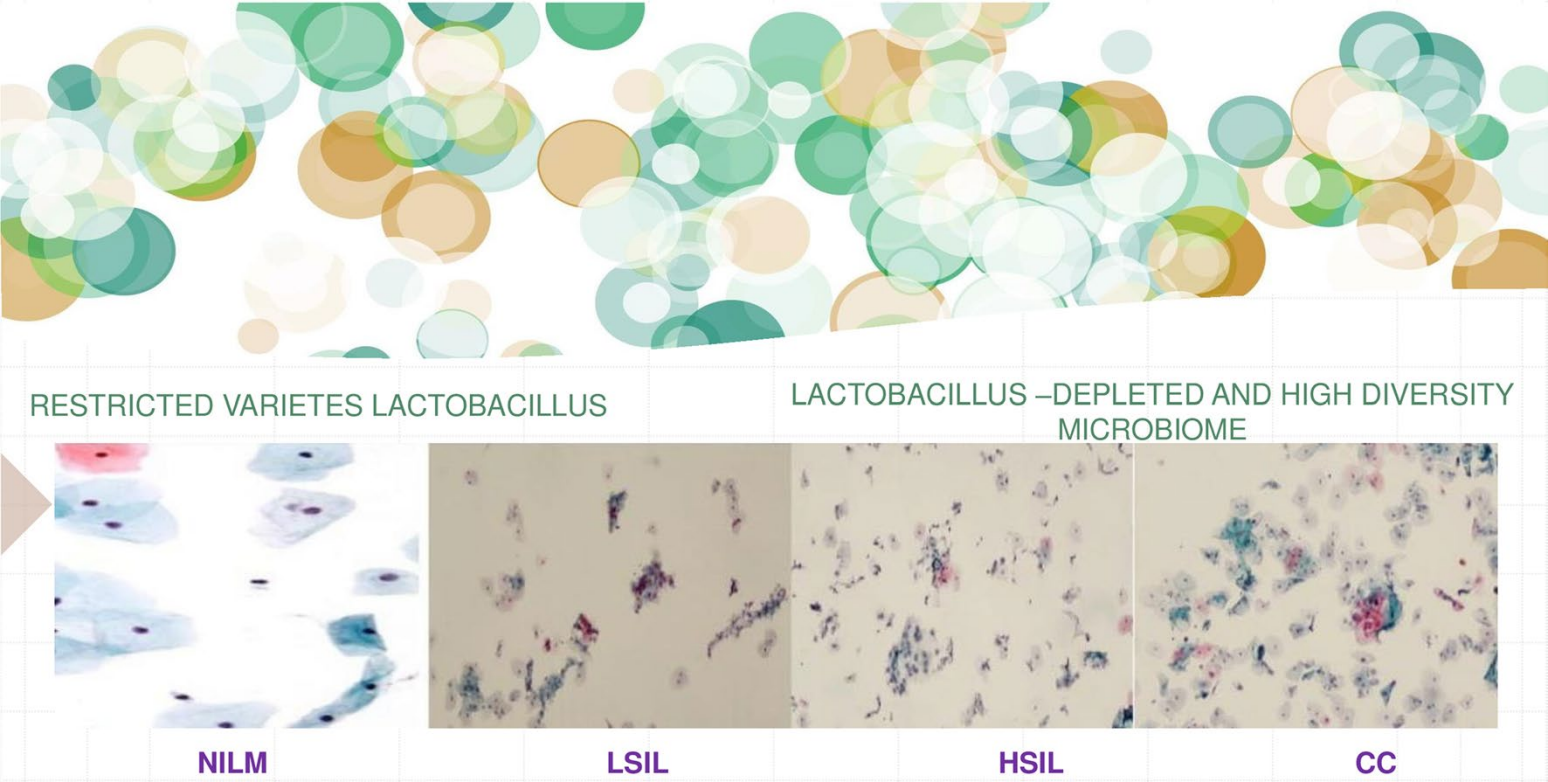
Vaginal microbiota and correlation with risk of cervical dysplasia. *NILM* negative for intraepithelial lesion or malignancy, *LSIL* low grade squamous intraepithelial lesion, *HSIL* high grade squamous intraepithelial lesion, *CC* cervical cancer

The vaginal microbiota in healthy women (eubiosis) contains a diversity of anaerobic and aerobic microorganisms. Lactobacilli are the most common, principal subpopulation. In selected situations, this balance can deteriorate (dysbiosis) and other pathogens may develop, decreasing the host anti-bacterial defenses [1]. The loss of this balance can lead, depending on several factors (hormonal levels, host defenses, sexual practices), to the onset of vaginosis, making the subject more susceptible to sexually transmitted diseases. Indeed, the maintenance of a healthy vaginal environment could be protective against infections [2]. The most common microbial species that populate the vagina (such as Lactobacilli) are the main protagonists in this process. Lactobacilli produce antimicrobial compounds (hydrogen peroxide, lactic acid, bacteriocin-like substances) and compete for adhesion sites in the vagina with other pathogens [3]. For this reason, a relationship between long lasting dysbiosis and cancer progression has been observed [4]. Indeed, through the elevation of pH, vaginal dysbiosis could promote cervical Human Papilloma virus (HPV)-related alterations and thus precancerous lesions progression [5]. HPV infection is among the most diffuse sexually transmitted infections in the world, with the highest incidence among young women, and has been recognized as the principal reason for the onset of cervical cancer [6]. The synergistic effects of persistent high-risk HPV infection, changes in the cervical microenvironment, and other carcinogenic factors (smoking, sexual propinquity) could favor the development of cervical precancerous lesions [7]. It appears that the composition of the microbiota may play a key role in conditioning individual susceptibility to infection and viral progression [8] (Fig. 1). Increasing attention is being devoted to probiotics, since they could promote women’s well-being by restoring the microbiota [9]. The aim of our analysis was to examine whether oral long term administration of *Lactobacillus crispatus* M247 in patients with HPV infections could restore eubiosis and consequently achieve viral infection control [10, 11]. The oral use of *Lactobacillus crispatus* M247 in order to modify the microbiota and increase HPV clearance, was recently evaluated [12]. *Lactobacillus crispatus* M247 is a tested probiotic for oral administration shown to have fecal and vaginal colonizing properties [13, 14]. We administered this strain in a preliminary, longitudinal, controlled, open study enrolling HPV-positive women, to assess its possible impact in terms of both the microbiota and of HPV status. The aim of this study was to assess the possible impact of oral long-term administration of *Lactobacillus crispatus* M247 on cervical cytology and the persistence of HPV infection. According to our experience, albeit partial, oral administration of the *Lactobacillus crispatus* M247 strain is a safe treatment and has a real potential to achieve the regression of PAP-smear alterations.

### Why was *Lactobacillus crispatus* M247 chosen for this study?

*Lactobacillus crispatus* M247 is a well-documented probiotic for oral administration that demonstrates fecal and vaginal colonizing properties. The rationale for the use of *Lactobacillus crispatus* M247 in HPV-infected women are the evidences showing that that *Lactobacillus crispatus* is detected more recurrently in patients without human papillomavirus (HPV) infection [10–13].

### Why did we prefer oral probiotic administration?

The administration of oral lactobacilli is based on the evidence that the intestinal and vaginal microbiota are linked, and that most vaginal dysbiosis may originate at the intestinal level [15–17]. Particularly, two recent studies investigated the relationship between the gut microbiome and cervical cancer [17]. Wang et al. compared the gut microbiome between eight women with cervical cancer and five healthy women [17]. Patients with cervical cancer showed a higher gut microbiota diversity, although this difference was not statistically significant [17]. However, in a larger examination of the gut microbiome in cervical cancer patients versus women without cervical cancer, our group observed a statistically significant higher diversity in cervical cancer patients than in healthy controls [18]. Moreover, literature reports have described significant differences in several taxa between cervical cancer patients and controls, predominantly superior numbers of members of the Proteobacteria phylum in women with cervical cancer [19]. Different studies reported distinct taxonomic abundance profiles between cervical cancer patients and the control group [18]. The intestinal microbiome is strongly conditioned by diet, as well as by individual factors. Therefore, Sims et al. concluded that since cervical HPV lesions are associated with vaginal and gut microbiota [20], oral probiotic treatment could modulate both microbiotas, reducing HPV lesions [20]. The use of oral *Lactobacillus crispatus* promotes colonization in the intestine that, in a few days [20] allows the transfer of lactobacilli into the vagina. This creates an intestinal reservoir of *Lactobacillus crispatus* that feeds the vaginal compartment even when the probiotic is no longer taken for a few weeks/months, which would not be possible after only topical application of the product. Thanks to the adhesion properties of *Lactobacillus crispatus* M247, this probiotic can long remain within intestinal and vaginal cells [21].

## Materials and methods

This is a pilot study, performed between February 2021 and February 2022 at the Department of Gynecological Obstetrics “San Paolo “of Bari. The initial study group included 205 sexually active women, aged between 30 and 64 years old, with a Papanicolaou test reporting low-grade cervical cytology in the form of atypical squamous cells of undetermined significance (ASCUS) or low-grade squamous intraepithelial lesions (L-SIL) and with positive HPV-DNA test. Exclusion criteria were pregnancy or breastfeeding, CIN2–3, immunological diseases or corticosteroid treatment, severe comorbidities. Patients were enrolled after a detailed explanation of the study aims, and underwent routine follow-up. Of the 205 patients selected according to the inclusion criteria, 160 women finally took part; features of the enrolled patients are summarized in Table 1. Each woman gave written informed consent to take part in the study and to publication of the results and completed all the necessary paperwork related to privacy. All women's data were completely anonymized, and the study was performed in accordance with the Declaration of Helsinki. Patients were randomized to two groups. Group 1 (n = 80) underwent oral treatment with *Lactobacillus crispatus* for 12 months (range 9–14 months). Group 2 (n = 80) constituted the control group that undergoing only routine follow-up every six months. The wet mount microscopy, PAP-smear and colposcopy were performed for every enrolled patient every 6 months, while the HPV-DNA test was repeated after 12 months. As literature reported, wet mount microscopy reflects the functional vaginal lactobacillary flora better than the Gram stain [11]. Lactobacillary grades (according to Donders' score), signs of bacteria, and presence of potential microbial balance alteration were documented [11]. In cases of infection, standard antibiotic or antifungal therapy was administered and at the end of therapy the bacteriological examination was repeated. Only after eradicating the infection were PAP smears performed. In addition, assessment of pH was made with the Litmus paper test. PAP-smears were assessed by expert pathologists by means of the three-tiered cervical intraepithelial neoplasia (CIN) classification and employing Bethesda terminology [9]. An adequate smear was defined as a sample with an adequate number of squamous cells, noting any evidence of transformation zone. Colposcopy assessment was executed employing the terminology presented by the Nomenclature Committee of International Federation for Cervical Pathology and Colposcopy in 2011 [12], identifying as Grade 1 (minor changes) the existence of a fine mosaic, fine punctate, a thin aceto-white epithelium, or an irregular geographic border and as Grade 2 (major changes) the presence of a sharp border, an inner border sign, a ridge sign, a dense aceto-white epithelium, coarse mosaic/punctate, or cuffed crypt openings. Atypical vessels or other suspicious signs of invasion (such as fragile vessels, irregular surface, exophytic lesions, necrosis, ulceration, tumor, or gross neoplasm) were included under Grade 2. The squamo-columnar junction visibility was classified as completely visible or non-completely visible. The transformation zone type was distinguished as type 1 and 2 (completely visible) or type 3 (not fully visible). The lesion position was described as: a) ectocervical (if the lesion was only present on ectocervical biopsy), endocervical (if the lesion was present on endocervical curettage), or ectocervical and endocervical (if the lesion was present on both).

**Table 1:** Features of HPV-positive patients enrolled in the randomized study

Number of partecipans	160
Nullipara	60/160 (37.5%)
Pluripara	100/160 (62.5%)
Monogamous heterosexual relationships	124/160 (77.5%)
Same-sex relationships	36/160 (22.5%)
Smoking	72/160 (45%)
Contraceptive pill use	38/160 (23.75%)
Symtoms of vaginosis	43/160 (26.8%)
Age (median-years)	45 (30–64)
Patients with high-risk HPV	105/160 (65%)
High HPV genotype	HPV 16: 29/105 (27.6%)
	HPV 18: 17/105 (16.1%)
	HPV 66: 13/105 (12.3%)
	HPV 68: 15/105 (14.2%)
	HPV 58: 10/105 (9.5%)
	HPV 45: 18/105 (17.1%)
	HPV 53: 12/105 (11.4%)
	HPV 51: 18/105 (17.1%)
	HPV 52: 17/105 (16.1%)
	HPV 35: 18/105 (17.1%)
Patients with concomitant Low risk HPV	55/160 (34%)
Low HPV genotype	HPV 6: 23/55 (41%)
	HPV 11: 29/55 (52.7%)
	HPV 40: 3/55 (5.3%)

### Pap test, HPV DNA test

The Pap test was executed by collecting exo- and endocervical cells by means of an Ayre spatula and a cytobrush. The cells were successively streaked on a glass slide and spray fixed. Smears were classified according to the 2014 Bethesda System [22]. The test was carried out with a validated method for screening COBAS 4800 HPV-Test Real-Time PCR that detects the strains: HPV-HR16, 18, 31, 33, 35, 39, 45, 51, 52, 56, 58, 59, 68. In each report it was specified that the finding of a positive test is an indication of the presence of the virus in the sample examined, but not in itself a diagnosis of disease [23].

### Statistical analysis

In total, 160 women aged between 30 and 65 years, with low-grade cervical cytology in the form of atypical squamous cells of undetermined significance (ASCUS) or low-grade squamous intraepithelial lesions (L-SIL), or with a negative PAP-smear but positive HPV-DNA test for HPV infection, were selected for this study. For each patient the following parameters were recorded.

Bacteriological examination every 6 months, PAP-smear and colposcopy every 6 months, HPV-DNA-test every 12 months. The collected data were inserted into an Excel database (Microsoft, Redmond, Washington, USA) and subjected to Least Significant Difference Testing, set at *p* ≤ 0.05. All statistical analyses were performed using the PlotIT program Ver. 3.2 (Scientific Programming Enterprises, Haslett, MI, USA).

### Adherence to therapy, tolerability, and treatment side effects

Patients referred a greater than 98% adherence to therapy; they declared that the treatment was well tolerated, with no significant side effects. Only nausea was reported by 2 patients, that reported taking it in the morning, so we suggested taking it before meals and the disorder subsided.

### Tested product

The probiotic product used in our clinical study was Crispact® sachets. Each sachet contains no fewer than 20 billion colony forming units of *Lactobacillus crispatus* M247 (lMG-P-23257) The product is manufactured by Alfa Omega (Copparo, Ferrara, Italy) and traded by Pharmextracta SpA (Pontenure, Piacenza, Italy). The product was notified to the Italian Health Authorities in 2019 (March 1st) with notification number 115450.

## Results

In total, 160 women were included in the study. Each patient underwent fresh bacteriology (wet mount vaginal bacteriology) testing before the PAP test; signs of infection was elicited in 25% of group 1 patients (13% bacterial, 12% mycotic vaginosis) and 18.4% of Group 2 patients (bacterial 12% and 6.4% mycotic vaginosis). Moreover, 95% of patients with vaginosis in group 1 and 98% in group 2 also referred intestinal disturbances (irritable colon, colitis). Vaginosis was identified following Amsel criteria [14] and by the presence of a vaginal discharge together with distinctive symptomsPatients with concomitant infections in both groups were treated by standard therapy for infections, and only after therapy and a repeated negative bacteriological examination was the Pap-test performed, in order to reduce the potential interference of infections on the reading of the pap-test. Pap smear abnormalities were associated with HPV DNA positivity in 73.5 in group 1 and 75.8% of cases in group 2. Patients were sequentially randomized to two groups, as previously explained (Group 1, n = 80 and Group 2, n = 80). Compliance was very good, and all participants returned for all follow-up visits. Median follow-up time was 12 months (range 9–14 months); first follow-up was after six months. At six months, there were no statistically significant changes on cytological and HPV test results between the two groups: remission rates of HPV lesions were (Group 1 = 20%, and Group 2 = 18%). At the end of the study period, the likelihood of resolving HPV-related cytological anomalies was statistically significantly higher in *Lactobacillus crispatus* M247 long-term oral users (61.5 vs. 41.3%, *p* = 0.041) (Group 1, n = 80) versus the control group. (Group 2, n = 80). The most common HPV genotype was type 16, found in 27.6% of positives, followed by type 51, 45 and 35 (17.1%), type 18 and 52 (16.1%) and type 68 (14.0%). HPV genotype 66 was found in 12.3% and 58 (9.5%) of positives.

However, HPV-DNA negativity documented at the end of the study period, after a median follow-up of 12 months (range 9–14 months), was not significantly different (*p* = 0.034) in patients with only follow-up (9.3%) as compared to patients who took long term oral Lactobacilli (15.3%).

## Discussion

Cervical cancer is the most common neoplasia among patients in developing countries and the second most frequent female cancer worldwide [24]. Frequently, it develops through a sequence of premalignant lesions, described as different grades of CIN 1, 2 and 3 [22]; just a few women with CIN lesions undergo progression to invasive cancer. Moreover, although HPV is widespread, literature reports show that only a small number of women have a recurrent HPV infection and subsequently develop precancerous lesions [25]. Indeed, several studies have described spontaneous HPV clearance, estimated at approximately 20–30% of cases at three months, almost 50% at six months, and nearly 60–70% at one year [26]. Consequently, several studies report that over 90% of HPV infections and infection-induced lesions are transitory and resolve spontaneously [27]. The ability of each organism to eliminate the virus may depend on several factors, among which concomitant vaginal infections, a local microflora imbalance and immune response defects seem to be decisive [6]. The role of chronic inflammation due to vaginal infections and the development of pre-cancerous lesions was recently profusely debated in literature as a risk factor for cervical dysplasia [28, 29]. Indeed, it was demonstrated that the vaginal microbiota can play a crucial role in the maintenance of vaginal homeostasis, with a protective role against fungal, protozoal, bacterial and viral infections. A defensive action has also been found against the human papillomavirus. A healthy vaginal ecosystem is characterized by lactobacilli, that could modulate the local immune system and inflammation response [30], being able to control cell proliferation/apoptosis [10]. Indeed, hydrogen peroxide, lactate and bacteriocins released by lactobacilli could inhibit pathogens overgrowth in the whole urogenital tract, offering a vital support in achieving good reproductive and general health in women [31–33]. Therefore, the support of lactobacilli taken orally can help to restore the endogenous balance and strengthen the defenses against HPV, facilitating its clearance [33–35]. Other studies evaluated the role of oral probiotics in patients with HPV infection. An important bias of these experience was the enrollment of only women with a concomitant infection in course. The execution of pap-smears in women with infection could be unreliable since the criteria for an optimal cytology evaluation also include the absence of inflammation [35–38]. Moreover, interestingly, women who had vaginosis had an associated chronic intestinal disease. This confirms the choice to administer oral lactobacilli, since the intestinal and vaginal microbiota are linked. Therefore, it would be useful to create a multidisciplinary equipe (made up of Gastroenterologists, Nutritionists and Gynecologists) to find a balance of these different aspects, restoring vaginal and intestinal eubiosis. Moreover, in the literature there is evidence of the impact of diet on the microbiota [29, 39–42]. However, to our knowledge, no study has yet correlated a balanced diet with an improved microbiota, making subjects less predisposed to vaginocervical infections. It would be important to test this hypothesis through a personalized, multidisciplinary approach. Moreover, this aspect of the research can be further probed through the study of HPV genotype. Indeed, in the present study HPV genotype are assess through the use of DNA Polymerase Chain Reaction (PCR) in L1 region and reverse hybridization.

The incidence of HPV could vary by country [43]. Particularly, our study was conducted in reference center for the screening of cervico-carcinoma located in Puglia. In our territory there is a clear prevalence of some genotypes in particular (16, 31, 45 and 51) [43]. The present study revealed that HPV 16, followed by genotypes 51, 35, 45 were the most persistent. These results are different from the others of previous studies, which report a greater persistence of HPV 16 and 18 [44]. Furthermore, the evaluation of demographic-behavioral characteristics in association with HPV clearance would require a multivariate statistical analysis that could be pursued in the future, favoring the identification of a targeted and personalized treatment [45].

Indeed, even if the effectiveness of vaginal lactobacilli appears limited, the concomitant use of oral and vaginal lactobacilli could be considered to potentially obtain HPV negativity in less time and probably also to reduce recurrences [46, 47]. Consequently, further studies are warranted, comparing several arms, and with the concomitant use of oral and vaginal probiotics.

### Limitation of study

The results of our study appear to encourage the use of probiotics in patients with low-grade HPV lesions. Therefore, our data must be carefully evaluated, since in the HPV natural history, 60–70% of patients undergo spontaneous regression within 12 months [23]. Consequently, it is difficult to assess whether PAP-smear negativization was linked to the oral probiotics treatment or to the described high incidence of spontaneous viral clearance in low grade lesions. On the other hand, there was no significant difference in the HPV test result between the two groups after 12 months. Based on our study results, we may state that oral long-term administration of *Lactobacillus crispatus* M247 could improve the microbiota and consequently the cytology, while the efficacy on definitive viral clearance, demonstrated with the HPV test, has still to be fully explored. Therefore, even if the data are encouraging, further studies of larger samples are warranted to verify the effectiveness of long term therapy with a probiotic, such as the analysis of microbiota community state types. Moreover, in cases of persistence, the viral strain could be evaluated by viral genotyping.

## Conclusion

In this study, long term administration of oral *Lactobacillus crispatus* M247 showed a potential effect on resolving cervical abnormalities, through restoring the physiological vaginal balance. Indeed, the high percentage of clearance of PAP-smear abnormalities obtained with oral *Lactobacillus crispatus* M247 long term administration offers encouraging evidence. For further confirmation, a metagenomics analysis of the vaginal, cervical, and intestinal microbiota in HPV infected women should be executed. Consequently, our experience could be a starting point for further discussions on this topic.

## Data Availability

All the data in the manuscript have been archived and are available through the corresponding author.

## Abbreviations

HPV: Human papillomavirus
ASCUS: Atypical undetermined significance
L-SIL: Low-grade squamous intraepithelial lesion
H-SIL: High-grade squamous intraepithelial lesion
CIN: Cervical intraepithelial neoplasia
